# Monitoring the BTEX Volatiles during 3D Printing with Acrylonitrile Butadiene Styrene (ABS) Using Electronic Nose and Proton Transfer Reaction Mass Spectrometry

**DOI:** 10.3390/s20195531

**Published:** 2020-09-27

**Authors:** Wojciech Wojnowski, Kaja Kalinowska, Jacek Gębicki, Bożena Zabiegała

**Affiliations:** 1 Department of Analytical Chemistry, Faculty of Chemistry, Gdańsk University of Technology, 11/12 Gabriela Narutowicza Street, 80-233 Gdańsk, Poland; bozena.zabiegala@pg.edu.pl; 2 Department of Process Engineering and Chemical Technology, Faculty of Chemistry, Gdańsk University of Technology, 11/12 Gabriela Narutowicza Street, 80-233 Gdańsk, Poland; jacek.gebicki@pg.edu.pl

**Keywords:** 3D printing, electronic nose, BTEX, volatiles, proton transfer, mass spectrometry

## Abstract

We describe a concept study in which the changes of concentration of benzene, toluene, ethylbenzene, and xylene (BTEX) compounds and styrene within a 3D printer enclosure during printing with different acrylonitrile butadiene styrene (ABS) filaments were monitored in real-time using a proton transfer reaction mass spectrometer and an electronic nose. The quantitative data on the concentration of the BTEX compounds, in particular the concentration of carcinogenic benzene, were then used as reference values for assessing the applicability of an array of low-cost electrochemical sensors in monitoring the exposure of the users of consumer-grade fused deposition modelling 3D printers to potentially harmful volatiles. Using multivariate statistical analysis and machine learning, it was possible to determine whether a set threshold limit value for the concentration of BTEX was exceeded with a 0.96 classification accuracy and within a timeframe of 5 min based on the responses of the chemical sensors.

## 1. Introduction

The additive manufacturing market is showing an impressive acceleration, with a 52% annual growth in 3D printer volume sales [1]. This is in part due to the increasing popularity of consumer-oriented desktop fused deposition modelling (FDM) 3D printers. The concept of “personal manufacturing” is gaining momentum due to the increased applicability of 3D printing in production and manufacturing [2,3]. The ability to produce a component with complex geometry in rapid, cost-effective way lowers the market entry barriers for small companies and start-ups, with applications in e.g., medicine, production of consumer goods, toys, and novelty items [4]. The general awareness of the usefulness of this technology increased drastically at the onset of the 2020 SARS-CoV-2 pandemic, when 3D-printing enthusiasts joined *en masse* the efforts to increase the availability of personal protection gear and modifications of medical equipment [5,6]. The relative ease of operating a modern 3D printer, together with the availability of 3D-printable content spurs a demand beyond the early adopters. Over half a million units were sold in 2017 alone [1], with many making their way to households and classrooms, where proper ventilation standards are not enforced.

Consumer-oriented desktop 3D printers based on FDM technology use polymer materials, usually in the form of solid filament, which is melted as it is passed through a heated extruder and then solidifies upon deposition in strata to gradually form the desired object. The heating of polymer filaments during printing leads to the emission of particulate matter and volatile organic compounds (VOCs). However, while the former has been relatively well-researched [7,8,9], there is little data available regarding the qualitative and quantitative emission of VOCs during 3D printing, due to the difficulties with reliable, real-time monitoring of the complex gaseous mixtures emitted during the thermal degradation of polymer materials. Thus, despite the increasingly common and widespread household use of desktop FDM 3D printers, there are no cost-efficient tools available to assess the user’s exposure to potentially harmful volatile chemical compounds. This is of particular concern when considering the use of filaments made of acrylonitrile butadiene styrene (ABS) which is one of the most popular materials in FDM 3D-printing due to its being relatively cost-effective, easy to machine and fabricate. However, thermal degradation of ABS leads to the production of multiple volatile organic compounds which are not created during thermal degradation of other common thermoplastics [10,11]. It is vital to be able to assess the operator’s exposure to benzene, toluene, ethylbenzene, and xylenes (BTEX) generated from styrene due to them being irritating, toxic, or, in the case of benzene, carcinogenic (Group 1 human carcinogen following the The International Agency for Research on Cancer (IARC) classification) [12,13,14,15,16]. It was estimated that styrene accounts for more than 30% of the total VOCs emitted from ABS [11].

However, in order to give the user of the consumer-grade 3D printer a timely, binary indication of whether a threshold concentration value of BTEX compounds has been exceeded, the proposed solution has to be available for the fraction of the price of the 3D printer itself. To this end, chemical gas sensors could be used, as demonstrated in previous studies in which metal oxide semiconductor (MOS) and surface acoustic wave sensors were employed with good results to detect BTEX compounds in the air [17,18]. While the commercially available sensors lack the sensitivity and specificity necessary to directly measure the concentration of benzene in 3D printing fumes, their response signals to the complex mixture of VOCs could be indirectly linked to a reference concentration of a target analyte determined using other methods by means of machine learning techniques. In such a scenario the lack of selectivity of the gas sensors could be in fact leveraged by implementing the electronic nose concept [19]. The price criterion limits the applicability of photo-ionization detectors (PIDs) which are otherwise well-suited for real-time determination of the total concentration of VOCs [20], since, at the time of writing, the commercial, consumer-grade FDM 3D printers can be acquired for approx. 1000 USD. However, the application of other commercially available chemical sensors, i.e., metal oxide semiconductor (MOS) and electrochemical sensors, seems feasible in this use case [21].

This leads to another difficulty since such an implementation of chemical sensors for indirect monitoring of BTEX compounds in near-real-time requires corresponding reference values—time-resolved data on the changes in the concentration of volatiles during 3D printing. There have been several attempts at the quantitative determination of VOCs generated during thermal degradation of polymers and/or assessment of the potential negative impact of their presence on human health [9,11,22,23]. However, the commonly used thermal desorption-based techniques such as thermal desorption gas chromatography-mass spectrometry or thermal desorption gas chromatography coupled with a flame ionisation detector which have been previously used to determine the overall composition of ABS 3D-printing fumes, provide information on the total emission rate throughout the measurement, without the necessary time resolution. The latter was achieved using photoionisation detectors (PID); however, it was at the expense of qualitative information, as only the total concentration of volatile organic compounds was measured [22,24]. Instead, we propose to use proton transfer reaction time-of-flight mass spectrometry (PTR-TOFMS) for direct, highly sensitive, real-time determination of the emission of BTEX compounds. This technique is well-suited to the task at hand, since owing to the nature of the ionization mechanism the 3D printing fumes can be analyzed directly, without the use of carrier gas. The omission of the sample preparation stage reduces the risk of errors, and, perhaps most importantly, the mixing ratio of a wide range of chemical compounds can be simultaneously calculated based on the known reaction rate constants to enable time-resolved concentration monitoring [25].

In this short communication, we describe a proof-of-concept study aimed at testing the applicability of an array of low-cost electrochemical sensors for indirect monitoring of the emission of BTEX compounds during 3D printing using ABS. This is, to the best of our knowledge, the first attempt to characterize the emission of VOCs during FDM 3D printing in real-time using a direct-MS technique, and the first instance of using an electronic nose to a similar end.

## 2. Materials and Methods

### 2.1. Materials and Reagents

The analytical standard-grade solutions used for establishing the PTR-MS fragmentation pattern (toluene, benzene, ethylbenzene and styrene) were obtained from Sigma-Aldrich (Merck KGaA, Darmstadt, Germany). The e-nose calibration solution was prepared using ethanol (HPLC-grade, Merck KGaA, Darmstadt, Germany), isopropanol, hydrogen sulphide, and a stock 25% ammonia solution (Avantor Performance Materials–formerly POCH, Gliwice, Poland). The 1.75 mm 3D-printing filaments designated for professional use (ABS 702 Black, ABS 702 Yellow, ABS 702 Natural,) were obtained from Nebula Filaments (Stare Bystre, Poland). The filaments were kept in vacuum-sealed packaging at room temperature prior to the experiments.

### 2.2. Proton Transfer Reaction Mass Spectrometry

The real-time changes in the mixing ratio of the monitored volatile compounds during FDM 3D printing with ABS filaments were monitored using the PTR TOF 1000 Ultra (IoniconGmbH, Innsbruck, Austria) PTR-MS coupled with a time-of-flight detector. The E/N value was kept at 120 Td by adjusting the voltage in the drift chamber to 610 V. The sampled printing fumes from within the enclosure were introduced into the drift tube at 100 cm^3^ min^−1^. It was previously demonstrated that the gaseous mixture generated during 3D printing consists mostly of styrene (>50%), ethylbenzene and other BTEX compounds, aldehydes, and other VOCs (>30%) [10,20,26]. The concentration of BTEX compounds and styrene was determined based on the kinetics of the proton transfer reaction with the H_3_O^+^ ion. For more details on PTR-MS, the reader is directed to Ellis et al. [27]. The calculations were based on the following k reaction rates (10^−9^ cm^3^ s^−1^): benzene 1.97, toluene 2.12, ethylbenzene and xylene 2.28, styrene 2.33 [28], and on the air density corresponding to the ambient temperature of 20 °C maintained in the laboratory throughout the experiments. The abundancies of the monitored volatiles were corrected based on the fragmentation pattern established by introducing the headspace of standard solutions into the device in a dynamic mode, using clean air supply and the built-in mass flow controller to obtain a split when necessary. The fragmentation patterns obtained for standard solutions of benzene, toluene, ethylbenzene and styrene at E/N of 120 Td are listed in Table S1 from the Supplementary Materials. They contain the pseudomolecular ion and other products of ionization, and their corresponding ratios which were used to calculate the concentration of the monitored volatiles. The mass spectra were averaged every second and were recorded continuously throughout each measurement, with approx. 3300 spectra recorded per experiment. The spectra were recorded using IoniTOF v. 3.0.76 and processed using PTR-MS Viewer v. 3.3.9.1 (IoniconGmbH, Innsbruck, Austria).

### 2.3. Electronic Nose

The electronic nose measurements were conducted in parallel to the PTR-MS. The device was a prototype e-nose development platform with electrochemical sensors mounted in modular, replaceable chambers designed to easily tailor the array of sensors to the particular matrix and measurement conditions and to assess their applicability in targeted designs. The design and operation of the device are described in detail by Wojnowski et al. [29,30]. The device was equipped with an array of seven electrochemical sensors: SPEC 110-303, SPEC 110-102, SPEC 110-507, SPEC 110-601, SPEC 110-901, SPEC 110-205 (SPEC Sensors, Newark, CA, USA), and NH3 3E 100 SE (City Technology, Portsmouth, UK) (see Table S2). It was operated periodically throughout the experiment in order to avoid sensor saturation, with 10 evenly-spaced measurements per experiment and the last measurement terminating with the PTR-MS measurements at approx. 3300 s. A single measurement cycle consisted of a 100 s purging phase, during which the sensors were purged with the ambient air and the baseline was established, followed by 100 s of sampling the atmosphere within the 3D printer enclosure, and a subsequent 100 s purge with the ambient air which was sufficient to re-establish the baseline [30]. The sampling was carried out dynamically at 100 cm^3^ min^−1^. The temperature and humidity of the sampled gaseous mixture were monitored using the built-in sensors in order to adjust the response signals accordingly. The data points for each sensor were collected by establishing the average response signal of the last 10 s (steady-state) of the sampling mode in relation to the corresponding baseline signal of the purge mode. While the electrochemical sensors are in general less prone to sensor drift than e.g., MOS sensors, the change in the baseline response signal throughout the measurement campaign was monitored daily by analyzing the headspace of a standard mixture. It consisted of ethanol, isopropanol, hydrogen sulphide and ammonia (100 ppm, 100 ppm, 200 ppm and 10 ppm, *v/v*, respectively), and was placed in a 20 mL headspace vial, sealed with a cap, and incubated for 10 min at 40 °C. While no significant effect was observed, it should be noted that the measurements were conducted within a relatively short time-frame of 5 weeks. The response signals were converted using the built-in ADC, corrected for temperature and humidity, then exported as comma-separated-values using dedicated Python-based software.

### 2.4. 3D Printer

The FDM 3D printing was carried out using the Prusa i3 MK2S printer (Prusa Research a.s., Prague, Czech Republic) with a 0.4 mm nozzle. The test print was two low-poly Pikachu figurines (thingiverse.com/thing:376601) scaled uniformly to 40 mm in the *z* axis (see Figure S1). The figurines were printed with no infill, with top, bottom, and wall thickness set to 1.2 mm. A single figurine weighted approx. 2.3 g. The time of a single print run was approx. 50 min, including the time necessary for heating up and auto-calibration of the printer’s *z*-axis stepper motors. The layer thickness was set to 0.2 mm, print speed to 40 mm/s, and travel speed to 100 mm/s. The printing temperature was 240 °C as recommended by the filaments’ manufacturer, and the build plate was heated to 80 °C, with no additional adhesive used. The model was converted to gcode using Cura v.3.2.1 (Ultimaker, Utrecht, Netherlands).

### 2.5. Experimental Setup

**Figure 1:**
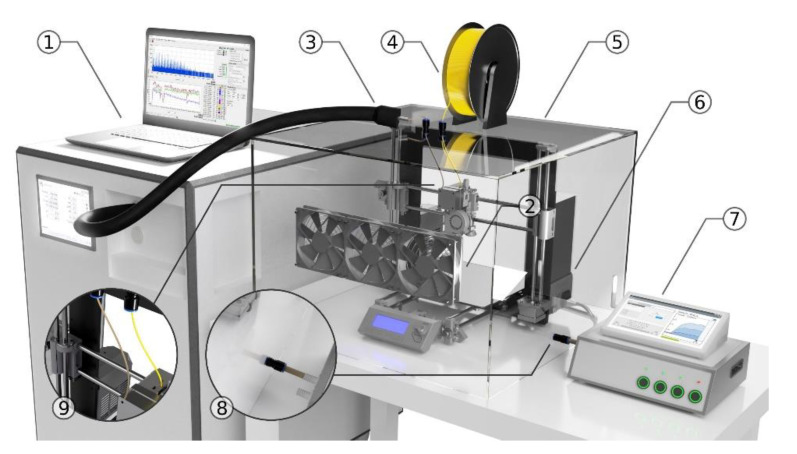
Experimental setup comprised of proton transfer reaction time-of-flight mass spectrometry (PTR-TOFMS) (**1**), 120 mm fans (**2**), polyether ether ketone (PEEK) capillary housed in a heated transfer line (**3**), 1.75 mm acrylonitrile butadiene styrene (ABS) filament (**4**), 3D printer enclosure (**5**), fused deposition modelling (FDM) 3D printer (**6**), and electronic nose (**7**), (**8**) and (**9**) show the positioning of the e-nose and PTR-TOFMS inlet capillaries, respectively.

The printer was placed in a 0.13 m^3^ enclosure, which is a common practice during FDM 3D printing, especially using ABS filaments due to the relatively high thermal shrinkage of this material which might result in the object detaching from the build plate mid-print as it cools unevenly. While in some previous studies much larger enclosures were used [31] which provide a good approximation of a scenario in which the user is exposed to emissions from a free-standing 3D printer, we have focused on a scenario in which the printer is enclosed. Usually, this is done using a dedicated enclosure supplied by the manufacturer of the 3D printer, in which case the volume of the enclosure might be smaller than the one used in the experiments, which was designed primarily to facilitate the sampling. The temperature within the enclosure increased during printing from 20 °C to 30 °C, and the relative humidity increased by an average of 6% relative to the ambient air. The enclosure was air-tight, but not hermetically sealed, with the main points of ingress being the small gaps in the PTFE access fittings for the filament and the PTR-MS capillary, and the egress through said capillary and through the electronic nose’s inlet capillary. The filament was placed outside of the enclosure in order to minimize its volume and to limit the generation of the volatiles originating from ABS to the effect of the printing itself. In order to evenly distribute the volatiles generated during printing within the enclosure, three standard 120 mm fans were mounted on its wall oriented towards the printer. The volatiles were sampled to the PTR-MS through a polyether ether ketone (PEEK) capillary with an inlet fixed on the printer head directly on top of the extruder motor. The sampled air was not filtered to limit the loss of analytes through adsorption. Outside of the enclosure, the capillary was housed in a transfer line at 70 °C. The detachable capillaries were rinsed with acetone, purged with synthetic air and incubated overnight at 80 °C after each measurement. The e-nose was placed next to the enclosure and connected to it with a short PTFE capillary. After each measurement, the 3D printer was allowed to cool to the ambient temperature and the enclosure was opened and vented until the raw signal corresponding to the *m*/*z* of the monitored compounds as observed using the PTR-MS returned to baseline (noise level). Since the 3D printer contains polymer elements (predominantly made out of PLA) which might have increased the background noise, a blank print measurement with no filament passed to the extruder was measured at the beginning of the experiment and after each filament change. Seven replicates for each of the three filaments were carried out, producing 210 multivariate e-nose data points and the corresponding real-time mass spectra. An overview of the experimental setup is shown in Figure 1.

### 2.6. Data Analysis

Data analysis was carried out using the Scikit-learn v. 0.23, LIBSVM v. 3.24 and Orange v. 3.25 Python packages [32,33,34]. The results of PTR-MS measurements were linked to the corresponding e-nose measurements by averaging the concentrations based on the *m*/*z* values characteristic for the target BTEX from the 10 s (10 consecutive mass spectra) matching the e-nose sampling periods described in Section 2.3. The response signals of the sensors were then normalized and evaluated based on their relevance for building a regression-based and binary classification models with the ReliefF algorithm [35], with the exceedance of the threshold limit value for the 79 Th ion characteristic for BTEX compounds used as the criterion in the latter. A stochastic gradient descent-based model (SGD) was used for regression, and both the SGD-based classifier and support vector machines (SVM) models were tested for classification. The SVM model involved the use of the RBF kernel (g = 0.33, that is 1/(number of features)). The SGD-based model used the squared ε intensive classification loss function and ridge (L2) regularization. For validation, the data were divided into three groups based on the filament, with results obtained from two filaments used for training and the results obtained from the third filament used for testing in order to test the robustness of the proposed approach. The PTR-MS data were processed as described in Section 2.2.

## 3. Results

### 3.1. PTR-MS Measurements

**Figure 2:**
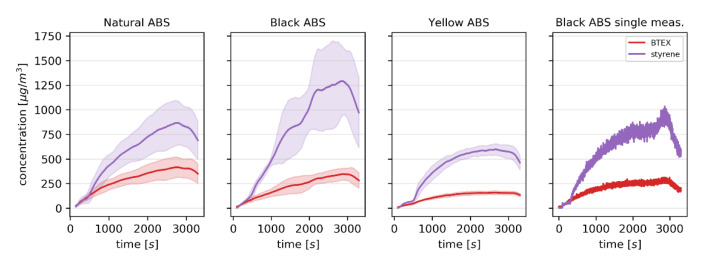
The concentration of styrene (purple) and the sum of *m*/*z* values characteristic for benzene, toluene, ethylbenzene and xylenes (red) in the 3D printer enclosure during printing with 3 different ABS filaments determined using proton transfer reaction mass spectrometry (PTR-MS). The solid line indicates the average ± standard deviation (shaded area). The final subplot illustrates data obtained during a single measurement.

The results of the PTR-MS monitoring of *m/z* values characteristic for BTEX compounds and styrene during printing with the three ABS filaments are shown in Figure 2.

The initial lag phase corresponds to the initial heating of the nozzle and of the build plate to the set temperatures, during which only the filament already within the nozzle undergoes thermal degradation. The concentration of the monitored VOCs within the enclosure then steadily increased until the end of printing at approx. 3100 s, at which point it decreased as the volatiles continued to be evacuated from the enclosure at 100 cm^3^ s^−1^ into the PTR-MS and periodically at an additional 100 cm^3^ s^−1^ during e-nose sampling. There are stark differences between the concentrations produced during printing with the three filaments, supporting previous reports in which the emission rates were higher in the case of natural ABS filaments compared to the filaments with coloring [36]. However, it is unclear to what extent this is caused by the effect of the coloring itself as opposed to the overall differences in the ratios of basic ingredients used to make the different filaments. The varying concentration and SD in the case of black ABS is the result of apparently greater thermal shrinkage and/or lower build plate adhesion of this filament which in some prints led to the detachment of the figurine from the build plate at approx. 1700 s and the accumulation of material at the nozzle. This, in turn, resulted in greater emission rates, as polymer other than just the deposited material was heated by the nozzle at a given time. This also likely caused the shift in the styrene/BTEX ratio, as the increase of temperature favors the formation of styrene oligomers and their subsequent cracking to release the monomers rather than the secondary reactions which lead to the formation of BTEX compounds [37]. To illustrate this effect, a single measurement in which the figurine detached from the build plate towards the end of the measurement, resulting in the rapid increase of styrene concentration and a much lower impact on the BTEX concentration, is also shown in Figure 2. This highlights the necessity of measuring the emission of potentially hazardous volatiles in real-time. The “detached” black ABS measurements were retained in the data set to test the robustness of the subsequently developed classification model.

**Figure 3:**
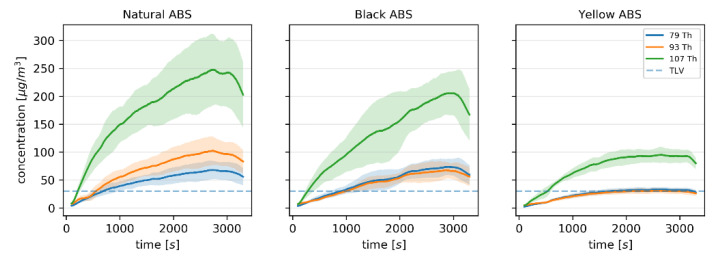
The concentration of ions characteristic for benzene, toluene, ethylbenzene, and xylenes (BTEX) compounds in the 3D printer enclosure during printing with 3 different ABS filaments determined using PTR-MS. The solid line indicates the average ± standard deviation (shaded area), and the dashed line indicates the threshold limit value (TLV) for the 79 Th ion at 30 μg m^3^.

The changes in the concentration of particular *m*/*z* values characteristic for BTEX compounds during printing were consistent with the changes in concentration of styrene, as shown in Figure 3.

Since it is difficult to discriminate between ethylbenzene and xylenes using direct-MS techniques such as PTR-MS based on their *m*/*z* value, and the ionization pattern of particular BTEX compounds (Table S1) hinders their exact determination, the *m*/*z* of 79 Th characteristic for these compounds was chosen as the reference data for the binary classification based on the response signals of the electrochemical sensors. A threshold limit value (TLV) was set to 30 μg m^3^ following the US EPA’s reference concentration for benzene inhalation exposure during a lifetime [38]. While the corresponding reference concentration for xylenes is set at 100 μg m^3^, the aforementioned difficulties with discerning between xylenes and ethylbenzene, and the fact that benzene is a known carcinogen, make the latter a better candidate for establishing a threshold criterion. It should be noted that the BTEX compounds are not the main constituents of the gaseous mixture generated during 3D printing with an ABS filament. While the TLV could be based on the concentration of styrene or other compounds if the proposed approach was used to monitor the emission of volatiles during printing using other polymers, the reason for focusing on BTEX compounds in the described study is the particular risk associated with the exposure to these volatiles.

### 3.2. Electronic Nose Measurements

**Figure 4:**
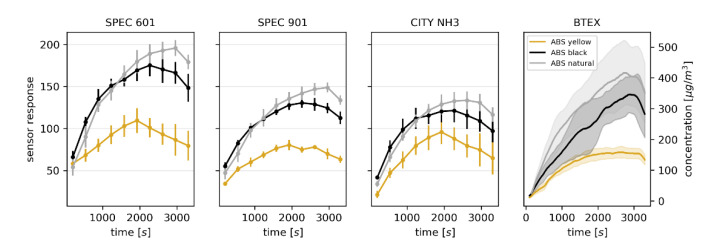
Average response signals of selected electrochemical sensors of the electronic nose to changes in the composition of the atmosphere within a 3D printer enclosure during printing with three different ABS filaments, and the corresponding BTEX concentration determined using PTR-MS. The error bars and the shaded areas correspond to ± SD.

Using the analysis of relevance it was determined that of the tested array of sensors, the SPEC 110-901, SPEC 110-601, and NH3 3E 100 SE sensors had the greatest impact on the classification based on the benzene TLV (ReliefF scores of 0.166, 0.129 and 0.095, respectively) (see Table S2). These sensors are nominally intended for monitoring respiratory irritants, SO_2_ and NH_3_, but it has to be stressed that they are only partially selective and their response signals cannot be treated as qualitative information when analyzing a complex gaseous mixture. As such, it is not surprising that their response signals do not closely follow the corresponding concentration values of BTEX compounds, as shown in Figure 4.

This resulted in the regression model having a R^2^ = 0.80 (Table S2). However, in the proposed application, i.e., binary classification based on the exceedance of the threshold value, the classification accuracy was 0.96, independent of the model. The ratios of the samples for which the reference value exceeded the TLV to the total number of samples were as follows: 0.33 for yellow ABS, 0.81 for black ABS, and 0.76 for natural ABS. When only the response signals of the SPEC 110-901 sensor (intended for monitoring respiratory irritants) were used, the overall classification accuracy was still satisfactory (0.94), except when models trained on black and natural ABS filaments were tested on the yellow filament, in which case the classification accuracy decreased to 0.78. This highlights the usefulness of the multivariate e-nose approach as opposed to using only a single sensor in the terms of robustness of the results. The confusion matrices for the training/testing validation are shown in Table S3.

## 4. Discussion

In this short communication, we described a concept study in which the changes of concentration of BTEX compounds within a 3D printer enclosure during printing with different ABS filaments was monitored in real-time using PTR-MS. These concentrations, in particular the concentration corresponding to the characteristic 79 Th ion, were then used as reference values for assessing the applicability of an array of low-cost electrochemical sensors in monitoring the exposure of the users of consumer-grade FDM 3D printers to potentially harmful volatiles. It was possible to determine whether the set threshold limit value was exceeded with a 0.96 classification accuracy and within a timeframe of a single measurement of 5 min. The results open two distinct avenues of future work. The first will be focused on obtaining a detailed characterization of the emission rate of volatiles through the thermal degradation of polymer filaments during deposition from 3D printer nozzles. In the described study, the experimental setup was designed to replicate the conditions in which the users operate the consumer-grade printers (in particular the enclosure encompassing the entire printer). A more fundamental study would involve confining the printer’s heated nozzle itself in a small-volume, hermetically sealed enclosure with a fixed flow of synthetic air passed through would allow taking full advantage of the temporal resolution of which the PTR-MS is capable, at the expense of real-life scenario implications. An even more comprehensive characterization of the emission rate and profile could be obtained by combining this approach with thermogravimetry.

The second, application-oriented direction is the development of a low-cost active sampling device with an array of chemical sensors, intended for alerting the users of FDM 3D printers of exposure risk. Here it should be noted that, while the sensor drift did not affect the measurements conducted during this study, and the used electrochemical sensors are overall not prone to suffering from it, it is nonetheless a significant factor which should be considered in such an implementation. The reported results were obtained within a relatively short timeframe of 5 weeks which is much shorter than the lifespan of a consumer-grade 3D printer. Furthermore, for such a device to be practical it needs to be user-friendly and, most importantly, available for a fraction of the price of the printer itself, which limits the feasibility of using elaborate calibration procedures. The availability and low cost of MOS sensor modules which can be easily integrated with the control boards of the enthusiast-grade 3D printers would make them a viable alternative to the electrochemical sensors used in this study.

## Supplementary Materials

The following are available online at https://www.mdpi.com/1424-8220/20/19/5531/s1, Figure S1: A 3D model of a low-poly Pikachu (thingiverse.com/thing:376601, left) and figurines printed out of yellow, natural and black ABS filament (right), Table S1: PTR-MS fragmentation pattern of selected compounds at E/N of 120 Td., Table S2: Sensors used in the electronic nose’s replaceable sensor modules and the corresponding ReliefF score for classification based on the exceedance of benzene TLV, Table S3: Confusion matrices for different training/testing groups and two different classification models. Results shown as percentage of actual.
