# Supplementary material for: Monitoring the BTEX Volatiles during 3D Printing with Acrylonitrile Butadiene Styrene (ABS) Using Electronic Nose and Proton Transfer Reaction Mass Spectrometry
Source: Sensors (Basel). 2020 Sep 27;20(19):5531. doi: 10.3390/s20195531 (PMC7582819; doi:10.3390/s20195531)
Supplement: Supplementary file 1 [file sensors-20-05531-s001.pdf]

sensors

Supplementary material

Monitoring the BTEX Volatiles During 3d Printing With Acrylonitrile Butadiene Styrene (ABS) Using Electronic Nose and Proton Transfer Reaction Mass Spectrometry

Wojciech Wojnowski 1,*, Kaja Kalinowska 1,*, Jacek Gębicki 2 and Bożena Zabiegała 1

[1] Department of Analytical Chemistry, Faculty of Chemistry, Gdańsk University of Technology, 11/12 Gabriela Narutowicza Street, Gdańsk, 80-233, Poland; bozena.zabiegala@pg.edu.pl
[2] Department of Process Engineering and Chemical Technology, Faculty of Chemistry, Gdańsk University of Technology, 11/12 Gabriela Narutowicza Street, Gdańsk, 80-233, Poland; jacek.gebicki@pg.edu.pl
* Correspondence: wojciech.wojnowski@pg.edu.pl (W.W.); kaja.kalinowska@pg.edu.pl (K.K.)

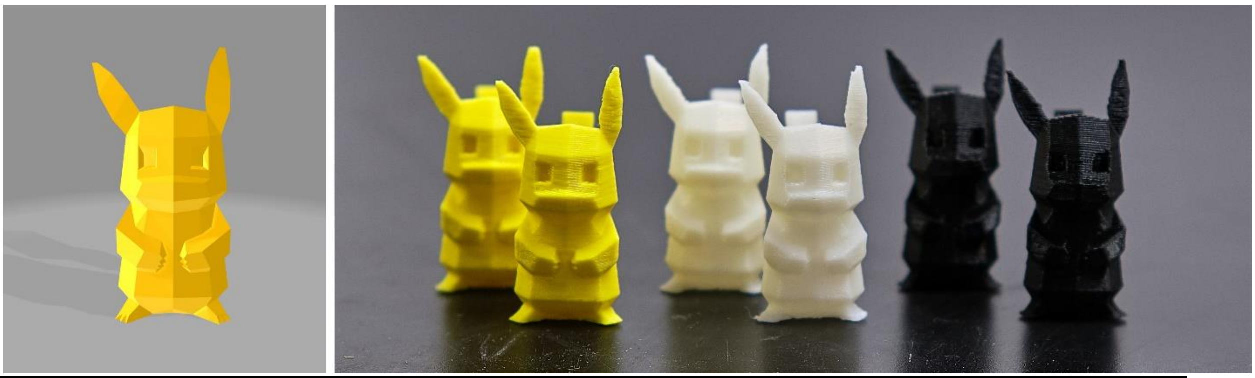

Figure S1. A 3D model of a low-poly Pikachu (thingiverse.com/thing:376601, left) and figurines printed out of yellow, natural and black ABS filament (right).

Table S1. PTR-MS fragmentation pattern of selected compounds at E/N of 120 Td.

Benzene		Toluene		Styrene		Ethylbenzene	
MH^+	ratio	MH^+	ratio	MH^+	ratio	MH^+	ratio
78.04	0.022	53.04	0.002	104.07	0.024	79.06	0.615
**79.06**	**0.917**	91.07	0.019	**105.09**	**0.905**	80.08	0.048
80.05	0.062	92.07	0.020	106.08	0.071	91.07	0.027
		**93.09**	**0.891**			**107.12**	**0.283**
		94.08	0.062			108.11	0.026
		95.06	0.006				

Table S2. Sensors used in the electronic nose's replaceable sensor modules and the corresponding ReliefF score for classification based on the exceedance of benzene TLV.

Sensor	Target analyte	Application	LOD [ppb]	ReliefF score	Spearman corr. with benzene conc.	Main cross-sensitivity
SPEC 110-507	NO_2	Air quality monitoring, industrial safety, air purification control	<20	0.048	0.34	Not provided
SPEC 110-601	SO_2	Air quality monitoring, industrial safety, air purification control	<20	0.157	0.86	Not provided
SPEC 110-303	H_2S	Industrial safety Monitoring, personal safety monitor, indoor and outdoor air quality, halitosis	<1000	0.094	0.74	NO_2, SO_2, Cl_2
SPEC 110-102	CO	Residential monitoring, ventilation control, indoor and outdoor air quality	500	0.091	0.80	H_2, isopropyl alcohol
SPEC 110-901	Respiratory irritants	HVAC ventilation control, indoor air quality, telemedicine, air purifier controls	<1000	0.199	0.89	H_2S, NO_2, SO_2, Cl_2
SPEC 110-205	EtOH	Breathalyzers	<1000	0.034	0.54	H_2S, CO, NO
CITY NH3 100	NH_3	Industrial safety	<1000	0.116	0.79	H_2S

Table S3. Confusion matrices for different training/testing groups and two different classification models. Results shown as percentage of actual.

Training / testing	Actual	Predicted			
		SVM		SGD	
		Above TLV	Below TLV	Above TLV	Below TLV
A*	Above TLV	92.7%	0.0%	92.7%	0.0%
	Below TLV	7.3%	100%	7.3%	100%
B**	Above TLV	98.2%	7.7%	94.9%	9.1%
	Below TLV	1.8%	92.3%	5.1%	90.9%
C***	Above TLV	75.0%	21.9%	100%	30.8%
	Below TLV	25.0%	78.1%	0.0%	69.2%

* training: black ABS, yellow ABS; testing: natural ABS.

** trainig: natural ABS, yellow ABS; testing: black ABS.

*** trainig: natural ABS, black ABS; testing: yellow ABS.
